# Biomimetic radiosensitizers unlock radiogenetics for local interstitial radiotherapy to activate systematic immune responses and resist tumor metastasis

**DOI:** 10.1186/s12951-022-01324-w

**Published:** 2022-03-04

**Authors:** Jiajia Zhang, Mengdie Yang, Xin Fan, Mengqin Zhu, Yuzhen Yin, Hongyan Li, Jie Chen, Shanshan Qin, Han Zhang, Kun Zhang, Fei Yu

**Affiliations:** 1 Department of Nuclear Medicine, Shanghai Tenth People’s Hospital, Tongji University School of Medicine, No. 301 Yan-chang-zhong Road, Shanghai, 200072 People’s Republic of China; 2 Institute of Nuclear Medicine, Tongji University School of Medicine, No. 301 Yan-chang-zhong Road, Shanghai, 200072 People’s Republic of China; 3 Department of Medical Ultrasound and Central Laboratory, Ultrasound Research and Education Institute, Shanghai Tenth People’s Hospital, Tongji University School of Medicine, No. 301 Yan-chang-zhong Road, Shanghai, 200072 People’s Republic of China

**Keywords:** Interstitial radiotherapy, Biomimetic radiosensitizers, Radiogenetics, PD-L1 upregulation, Hypoxic and immunosuppressive microenvironment

## Abstract

**Background:**

Similar to other local therapeutic methods, local interstitial radiotherapy (IRT) also suffers from insufficient systematic immune activation, resulting in tumor metastasis.

**Results:**

Mn-based IRT radiosensitizers consisting of ^131^I, MnO_2_ and bovine serum albumin (BSA) (^131^I-MnO_2_-BSA) were engineered. Such Mn-based IRT radiosensitizers successfully unlocked radiogenetics to magnify systematic immune responses of local IRT via remodeling hypoxic and immunosuppressive microenvironments and resist tumor metastasis. The MnO_2_ in ^131^I-MnO_2_-BSA caused decomposition of H_2_O_2_ enriched in tumors to generate O_2_ for alleviating hypoxic microenvironment and removing tumor resistances to IRT. Concurrently, hypoxia mitigation by such radiosensitizers-unlocked radiogenetics can effectively remodel immunosuppressive microenvironment associated with regulatory T (Treg) cells and tumor-associated macrophages (TAMs) infiltration inhibition to induce immunogenic cell death (ICD), which, along with hypoxia mitigation, activates systematic immune responses. More intriguingly, ^131^I-MnO_2_-BSA-enabled radiogenetics can upregulate PD-L1 expression, which allows anti-PD-L1-combined therapy to exert a robust antitumor effect on primary tumors and elicit memory effects to suppress metastatic tumors in both tumor models (4T1 and CT26).

**Conclusions:**

IRT radiosensitizer-unlocked radiogenetics and the corresponding design principle provide a general pathway to address the insufficient systematic immune responses of local IRT.

**Graphical Abstract:**

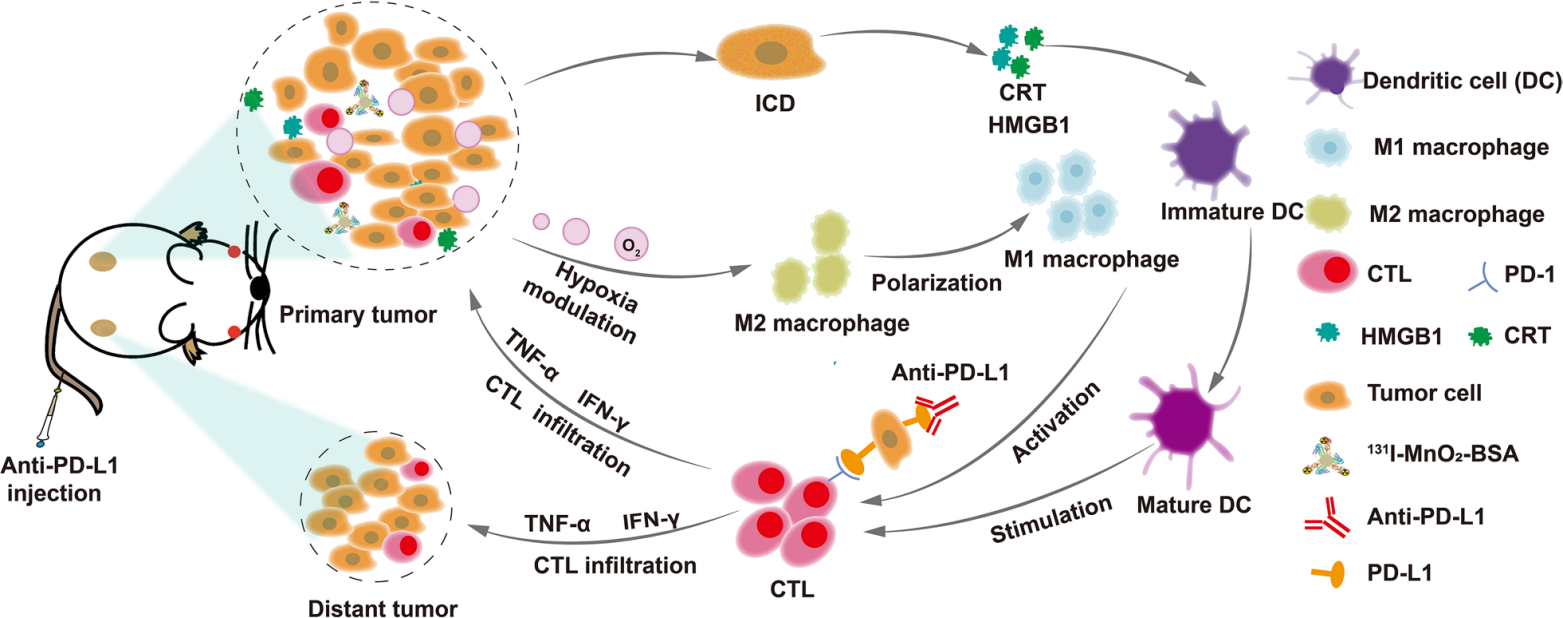

**Supplementary Information:**

The online version contains supplementary material available at 10.1186/s12951-022-01324-w.

## Background

Akin to ultrasound [1, 2], radiofrequency and laser [3, 4], interstitial radiotherapy (IRT) that serves as a local therapeutic modality has attracted increasing interest due to its precision, controllability and safety in comparison to external beam radiotherapy (EBRT). Nevertheless, IRT also bears the same drawback that other local treatment methods suffer from, i.e., insufficient systematic immune responses [5]. Consequently, inactivated systematic immune responses usually fail to suppress tumor relapse and metastasis even though IRT has been documented to activate local immune responses and promote immunogenic cell death (ICD) [6–8]. Given that, explorations on immune checkpoint blockade (ICB) therapy including anti-CTLA-4 and anti-PD/L1 was combined to inhibit or even eliminate metastatic tumors [9–12]. Despite a series of inspiring achievements in laboratory, ICB benefits only a small portion of patients in clinical practice [13]. This phenomenon is attributed to the failure of systematic immune response activation resulted from the ubiquitous hypoxic and immunosuppressive microenvironments that can aggravate the insufficient systematic immune responses [5, 14]. In detail, hypoxic microenvironment can elicit potent resistances to IRT-represented reactive oxygen species (ROS)-rooted antitumor methods and go against ROS production [15–17]. Moreover, hypoxia can further aggravate immunosuppressive microenvironment by recruiting a large number of immunosuppressive cells (e.g., regulatory T cells (Tregs) and tumor associated macrophages (TAMs)) to tumor and promoting immune escape, consequently inactivating the systematic immune responses and disabling immunotherapy [18–20]. Therefore, improving systematic immune responses after IRT to repress tumor metastasis by remodeling hypoxic and immunosuppressive microenvironments merits to be highlighted and is urgently demanded.

**Scheme 1:**
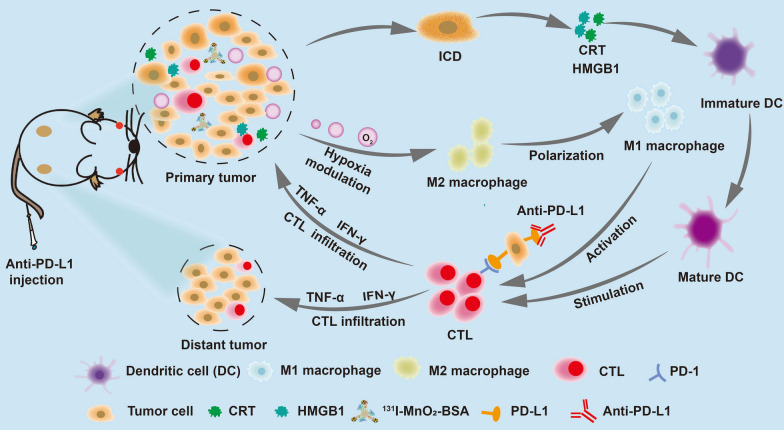
The schematic diagram and action mechanism of such radiosensitizers-unlocked radiogenetics in magnifying systematic immune responses including mitigating hypoxia, removing hypoxia-induced resistance to IRT, remodeling the immunosuppressive microenvironment and up-regulating PD-L1 for potentiating the anti-PD-L1 immunotherapy

In this report, we engineered an IRT radiosensitizer consisting of bovine serum albumin (BSA)-coated MnO_2_ (MnO_2_-BSA) and radionuclide ^131^I to unlock radiogenetics and enhance IRT-activated systematic immune responses against tumor progression and metastasis by remodeling hypoxic and immunosuppressive microenvironments and altering cancer cell phenotype (Scheme 1). In this IRT radiosensitizer, MnO_2_-BSA nanoparticles were yielded via a well-established biomimetic mineralization method, followed by radionuclide ^131^I labeling via substitution linkage due to the rich phenolic hydroxyl groups of BSA in ^131^I-MnO_2_-BSA [21]. MnO_2_ could trigger the decomposition of intratumoral H_2_O_2_ to generate O_2_ via the Fenton-like reaction, which enabled hypoxia mitigation and hypoxia-induced resistance liberation to favor IRT against malignancies [22–24]. More significantly, O_2_ release from ^131^I-MnO_2_-BSA nanocomposite ameliorated or even remodeled the immunosuppressive microenvironment by repressing Treg and TAM infiltrations and converting protumorigenic M2-type TAMs to antitumorigenic ones (Scheme 1), which allowed more cytotoxic T lymphocytes (CTLs) to enter tumors and come into action. Sequencing analysis also reflected that the hypoxic and immunosuppressive microenvironments mattered the enhanced IRT by such Mn-based radiosensitizers and their unlocked radiogenetics. Amazingly, this radiosensitizer-unlocked radiogenetics increased PD-L1 expression on the surface of tumor cells, which, provided an opportunity for combined therapy with PD-L1 blockade since high PD-L1 expression is the premise of highly-efficient anti-PD-L1 therapy [25]. By virtue of hypoxia mitigation, immunosuppressive microenvironment remodeling and PD-L1 upregulation-enabled therapy combined with anti-PD-L1 therapy, the IRT radiosensitizers are equipped with the most robust antitumor activity against primary and metastatic tumors in vivo, which holds high potential for clinical translation.

## Results and discussion

### Synthesis and characterizations of MnO_2_-BSA and ^131^I-tethered MnO_2_-BSA

**Fig. 1:**
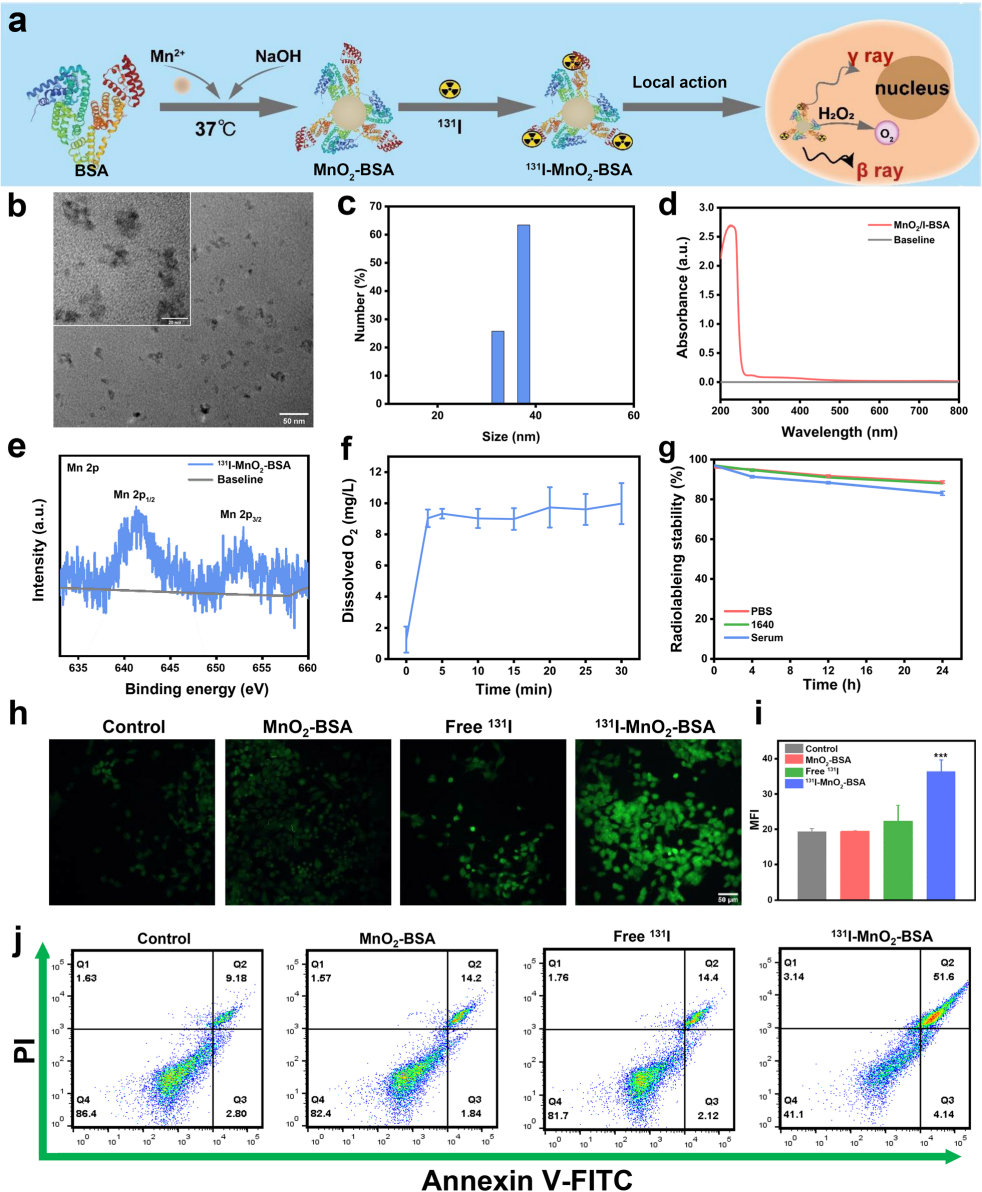
Synthesis and cellular-level evaluations of ^131^I-MnO_2_-BSA. **a** The synthesis and action procedures of radionuclide labeled MnO_2_-BSA (i.e., ^131^I-MnO_2_-BSA) and the enhanced IRT. **b** TEM images with low- and high- (inset) magnifications of MnO_2_-BSA; **c** Size distribution of ^131^I-MnO_2_-BSA determined by DLS in PBS. **d** UV–Vis absorbance spectra of ^131^I-MnO_2_-BSA with different concentrations in PBS. **e** Narrow-band XPS spectrum of ^131^I-MnO_2_-BSA, and Mn 2p XPS spectra with high resolution were recorded. **f** Oxygen dissolution level of ^131^I-MnO_2_-BSA after incubation with H_2_O_2_ (5 mM) for different durations. **g** Radiolabeling stability tests of ^131^I-MnO_2_-BSA at 37 °C in PBS, FBS and serum. **h**, **i** Fluorescence images (**h**) and the corresponding mean fluorescence intensity (MFI) (**i**) of ROS in 4T1 cells stained with DCFH-DA after various treatments. **j** Flow cytometry (FCM) patterns of 4T1 cells stained with propidium iodide (PI) and annexin V-FITC after various treatments for determining the apoptosis level. P values were calculated by ANOVA (***p < 0.001). Data are expressed as mean ± standard deviation (SD) (*n* = 3)

MnO_2_-BSA and ^131^I-MnO_2_-BSA were synthesized via an environmentally friendly biomimetic mineralization method (Fig. 1a) [26]. Uniformly-distributed MnO_2_-BSA nanoparticles with an ultrasmall size of approximately 10.0 nm are observed (Fig. 1b). Their hydrated dynamic size remains below 50 nm, as determined in dynamic light scattering (DLS) data (Fig. 1c). The ultrasmall size is probably attributed to the BSA-arised confinement effect that can impede MnO_2_ overgrowth and coincidently avoid aggregates’ birth [27, 28]. An evident characteristic absorbance peak at 200–300 nm corresponding to MnO_2_ in the UV–Vis spectrum of MnO_2_-BSA indicates the successful synthesis of MnO_2_-BSA (Fig. 1d). Wide-band and narrow-band X-ray photoelectron spectroscopy (XPS) analysis also reflect the presence of MnO_2_, wherein the valence of Mn is determined to be + 4 (Fig. 1e and Additional file 1: Fig. S1), and Mn^4+^ indeed favors O_2_ release after the catalytical decomposition of H_2_O_2_ (Fig. 1f). Radionuclide ^131^I labeling was tethered to BSA via a substitution linkage due to the rich phenolic hydroxyl groups of BSA [21], putting the final product (^131^I-MnO_2_-BSA) within easy reach. Moreover, the IRT radiosensitizers (i.e., radiolabeled ^131^I-MnO_2_-BSA) feature prolonged radiolabeling stability under different physiologic conditions at 37 °C, which offers enough time for the following studies (Fig. 1g). Despite losing 20%, the residual radiolabeling stability at a plateau of 80% after 24 h will be adequate for killing tumor, during which the structural stability without BSA shedding is reached due to no evident particle size variation (Additional file 1: Fig. S2).

### In vitro antitumor evaluations using ^131^I- MnO_2_-BSA

The viability of 4T1 cells treated with MnO_2_-BSA remains 80% even when Mn concentration reaches 90 μM (Additional file 1: Fig. S3a), suggesting that MnO_2_-BSA can serve as a safe carrier of ^131^I. Moreover, due to hypoxia-induced resistance to IRT, the ^131^I radionuclide alone fails to kill 4T1 cells (Additional file 1: Fig. S3a). Excitingly, the combination of ^131^I radionuclide and MnO_2_-BSA (i.e., ^131^I-MnO_2_-BSA) receives a significantly-elevated ability to kill 4T1 cells. This phenomenon is probably attributed to that MnO_2_-BSA engulfment by 4T1 cells, allowing a large amount of ^131^I to enter 4T1 cells and simultaneously give rise to O_2_ for mitigating hypoxia and liberating the hypoxia-induced imprisonment to IRT, both of which cooperatively contributed to the augmented ROS production. To verify this hypothesis, intracellular ROS that play a significant role in cell apoptosis and proliferation suppression are detected [29], wherein 2,7-dichloro-dihydrofluorescien diacetate (DCFH-DA) was used as ROS indicator [30, 31]. As expected, ^131^I-MnO_2_-BSA performs the best in provoking ROS birth (Fig. 1h,i and Additional file 1: Fig. S3b), consequently receiving the highest cell apoptosis, as evidenced by flow cytometry (FCM) inspection (Fig. 1j).

### In vivo O_2_-enhanced radioisotope therapy enabled by ^131^I-MnO_2_-BSA-unlocked radiogenetics

**Fig. 2:**
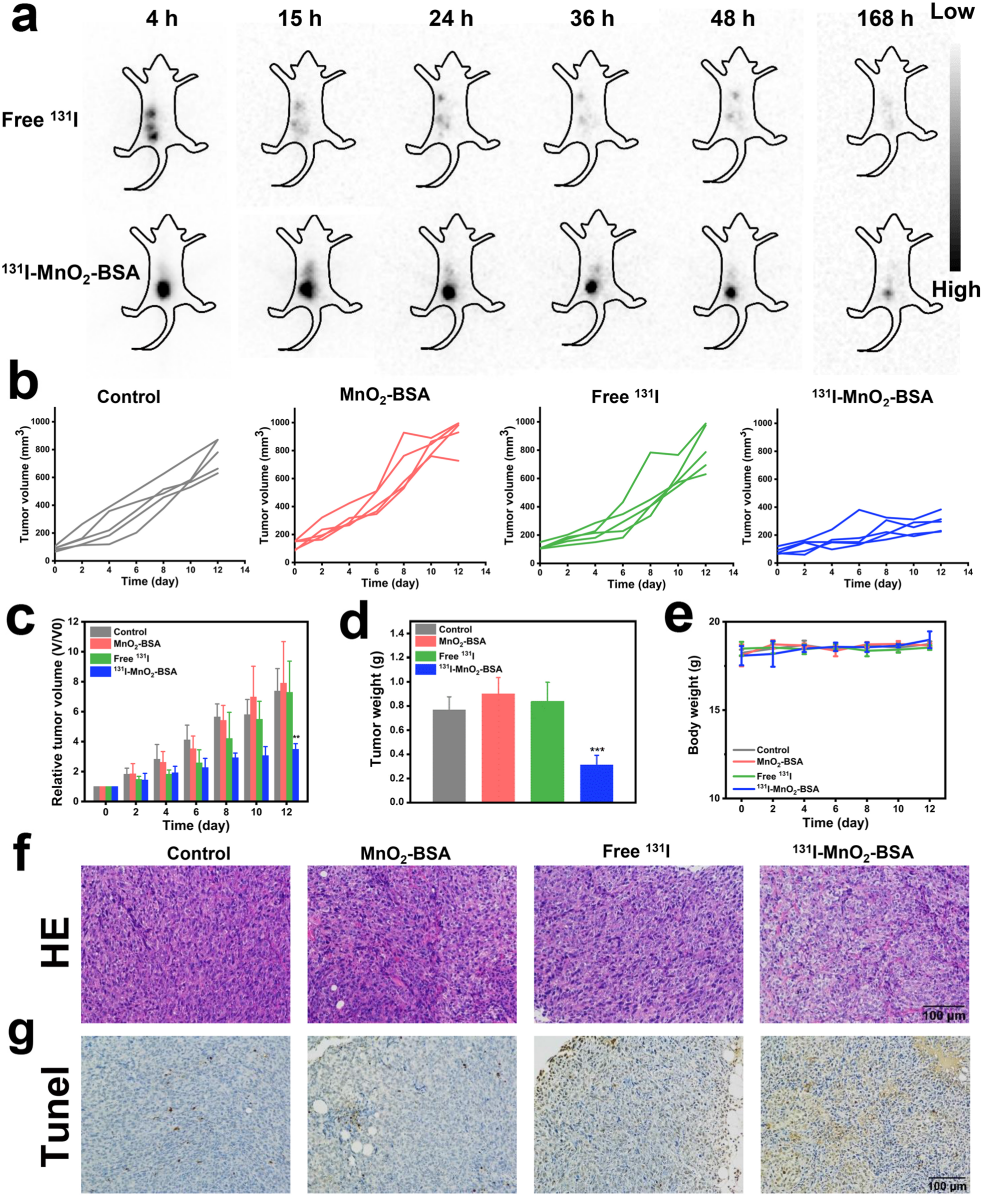
In vivo SPECT/CT imaging and antitumor evaluations using ^131^I-MnO_2_-BSA on 4T1-bearing mice model. **a** Representative SPECT/CT planar imaging of 4T1 tumor-bearing mice post-*i.t.* injection of ^131^I-MnO_2_-BSA at different time points, dose: 500 µCi; **b** Tumor growth curves of each mouse in different groups such as control, MnO_2_-BSA, free ^131^I and ^131^I-MnO_2_-BSA (dose: 500 µCi). **c** Time-dependent relative tumor volume variations of 4T1 tumor-bearing mice experiencing corresponding treatments in different groups including control, MnO_2_-BSA, free ^131^I and ^131^I-MnO_2_-BSA (dose: 500 µCi), where tumor volumes were normalized to initial values (V/V0). **d** Average tumor weight harvested from 4T1 tumor-bearing mice experienced different treatments in different groups at the end of experimental period (Day 12). **e** Body weight variation of 4T1 tumor-bearing Balb/c mice during treatment. **f**, **g** Optical microscopic images of H&E (**f**) and TUNEL-stained (**g**) tumor sections in different treatment groups (e.g., control, MnO_2_-BSA, free ^131^I and ^131^I-MnO_2_-BSA). P values were calculated by ANOVA (**P < 0.01 and ***P < 0.001). Data are expressed as mean ± SD (*n* = 5)

Inspired by above appealing therapeutic results in vitro, a subcutaneous 4T1 tumor-bearing mouse model was established to map the metabolic distribution of free ^131^I and ^131^I-MnO_2_-BSA radiosensitizers after local injection. Planar images were captured at different time intervals, and most of free ^131^I escaped rapidly from the tumor site, indicating low tumor retention. On the contrary, high ^131^I-MnO_2_-BSA accumulations at the tumor site without obvious diffusion into normal organs are observed even at 168 h (Fig. 2a), indicating that labeling with MnO_2_-BSA chelation can deliver ^131^I radioisotope and prolong the residence time available for ionizing irradiations. Notably, although the agent was intratumorally injected, a small proportion inevitably permeated into the circulation and entered stomach.

Thereafter, we evaluated the IRT efficacy of labeled ^131^I-MnO_2_-BSA in local tumors. In detail, mice bearing subcutaneous 4T1 tumors were intratumorally injected with MnO_2_-BSA, free ^131^I (*i.t.*, 500 μCi), ^131^I-MnO_2_-BSA (*i.t.*, 500 μCi). Tumor growth curves show that MnO_2_-BSA fails to delay tumor progression at the administered dosage (Fig. 2b, c). Additionally, free ^131^I is also disabled to delay tumor growth because of its rapid clearance or metabolism. Intriguingly, once ^131^I is combined with MnO_2_-BSA, the obtained ^131^I-MnO_2_-BSA is found to significantly suppress tumor growth (Fig. 2b, c). In detail, the tumor growth treated with ^131^I-MnO_2_-BSA at 12 days post-treatment is retarded, showing a 53% decrease in comparison to that in the control (Fig. 2c). Identical results are obtained by measuring tumor weight at the end of the experimental period, wherein the tumors of mice treated with ^131^I-MnO_2_-BSA weigh much less than the tumors from the mice in the other groups (Fig. 2d).

Additionally, no abnormal body weight loss or cachexia in the various treatment groups indicates that the nanomaterials have no latent short-term toxicity, which is promising for clinical applications (Fig. 2e). Pathological examinations including hematoxylin and eosin (H&E) staining and terminal deoxynucleotidyl transferase dUTP nick end labeling (TUNEL) assays, were performed to explored apoptosis and/or necrosis. H&E results show that the tumor cells in monotherapy (MnO_2_-BSA alone or ^131^I alone) groups retain relatively intact structure, while the tumor cells in ^131^I-MnO_2_-BSA group feature cell shrinkage, karyolysis, and nuclear fragmentation (Fig. 2f). Consistently, TUNEL assay further confirms these results, wherein the combined therapy, i.e., ^131^I-MnO_2_-BSA, brings about the most cell apoptosis (Fig. 2g).

### RNA-seq analysis for monitoring gene mutations arising from ^131^I-MnO_2_-BSA-unlocked radiogenetics

**Fig. 3:**
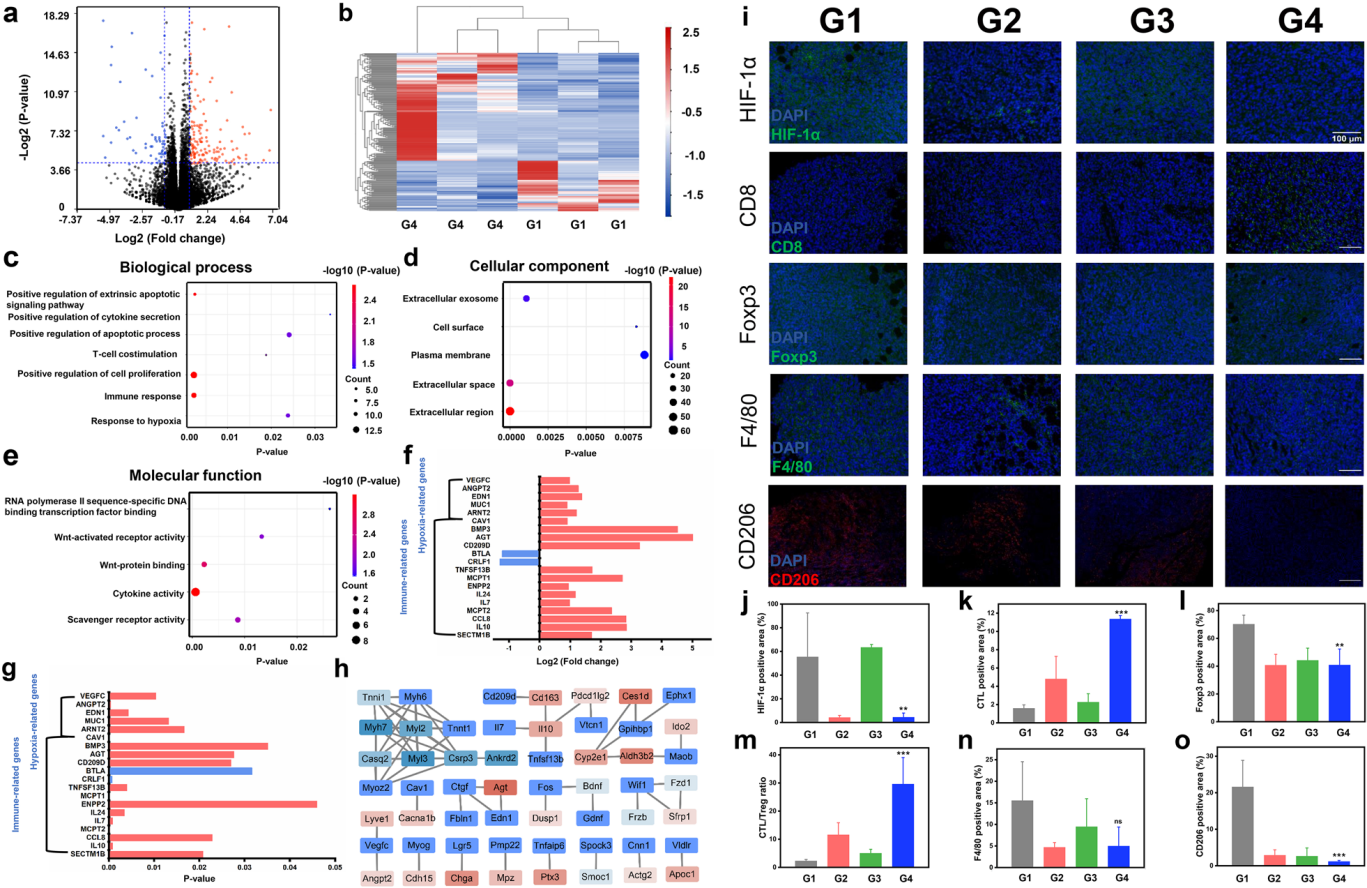
RNA-sequencing analysis, hypoxia modulation and systematic immune response activation of local ^131^I-MnO_2_-BSA treatment in 4T1-bearing mice model. **a** Volcano plots showing the DEGs between the control and ^131^I-MnO_2_-BSA groups; **b** Heatmap of DEGs between the control and ^131^I-MnO_2_-BSA groups; **c** Biological process analysis; **d** Cellular component analysis; **e** Molecular function analysis. **f** The Log2 fold change of DEGs related to immune and hypoxia response; **g** The p value of DEGs related to immune and hypoxia response; **h** Protein–protein interaction networks. G1 and G4 represent Control, ^131^I-MnO_2_-BSA, respectively. **i** Immunofluorescence images of tumors slices in different groups, and tumor slices were harvested from those mice experiencing corresponding treatment in different groups, and then stained with anti-HIF-α, anti-CD8, anti-Foxp3, anti-F4/80, and anti-CD206 antibodies, respectively. **j**–**o** Statistical data of HIF-1α+ (**j**), CTLs+ (**k**), Foxp3+ (Treg, **l**), CTLs/Treg (**m**), F4/80+ (**n**) and CD206+ (**o**) areas in tumors after various treatments, which were obtained from **i**. The ratios were normalized in relation to the PBS group. P values were calculated by ANOVA (***p < 0.001; *p < 0.05; ns, not significant). G1–G4 represent Control, MnO_2_-BSA, free ^131^I and ^131^I-MnO_2_-BSA treatment, respectively. Data are expressed as mean ± SD (*n* = 3), dose: 500 µCi

To determine the antitumor response mechanism of locally-applied ^131^I-MnO_2_-BSA from the genetics perspective, RNA-seq was performed to compare the differences in gene expression among tumor-bearing mice. Compared with the control group, 197 genes are identified as differentially expressed genes (DEGs) with a cutoff of |log_2_FC|= 0.9, among which 135 genes are up-regulated and 62 genes are down-regulated, as indicated in the volcano plot and heatmap plot (Fig. 3a, b). To gain further insights into the potential mechanisms of these DEGs, gene ontology (GO) analysis including biological process (BP), molecular function (MF) and cellular component (CC), was adopted. In the BP analysis, “positive regulation of extrinsic apoptotic”, “signal pathway positive regulation of cytokine secretion”, “T-cell co-stimulation, immune response”, and “response to hypoxia” are significantly affected by ^131^I-MnO_2_-BSA treatment (Fig. 3c). CC analysis demonstrates that DEGs participate in “extracellular exosome” and “extracellular region” (Fig. 3d). For MF, the DEGs mainly correlate with “cytokine activity” and “scavenger receptor activity” (Fig. 3e). In particular, the Log2 fold change and P value of the DEGs involved in immune-related pathways and hypoxia-related pathways were measured. Results show that the genes associated with immune-related pathways and hypoxia-related pathway including SECTM1B, IL10, CCL8, MCPT2, IL7, IL24, ENPP2, MCPT1, TNFSF13B, CD209D, AGT, BMP3, CAV1, ARNT2, MUC1, EDN1, ANGPT2, VEGFC, are up-regulated in ^131^I-MnO_2_-BSA treated mice, accompanied which mRNA expressions of CRLF1 and BTLA are down-regulated (Fig. 3f, g). Additionally, a protein–protein interaction (PPI) is obtained (Fig. 3h).

All of these sequencing results unveil that the Mn-based radiosensitizers-unlocked radiogenetics activated hypoxia and immune-underlined microenvironment to augment the antitumor affects. Therefore, to deeply understand it, tumor microenvironment was further surveyed as described in the following parts.

### ^131^I-MnO_2_-BSA for modulating the tumor hypoxic and immunosuppressive microenvironments

Hypoxia and immunosuppression are usually identified as the predominant factors that cause the failures of most antitumor treatment methods. Thus, numerous efforts have been made to modulate or even reverse these characteristics of the tumor microenvironments, remove treatment resistance and exert robust antitumor activities towards tumor progression, relapse and metastasis by releasing O_2_, elevating ROS half-life [15], breaking redox balance [32], blocking migration pathway [33], etc. MnO_2_-BSA is expected to mitigate the hypoxic microenvironment and liberate the hypoxia-induced imprisonment to IRT since Mn-based nanoparticles have been widely accepted to produce O_2_, produce massive ROS and even monitor treatment process via Fenton-like reactions, similar to Fe-based nanoparticles [23, 28]. To investigate how such IRT radiosensitizers-unlocked radiogenetics remodel hypoxic and immunosuppressive microenvironments, tumor microenvironments after different treatments were further investigated to analyze the antitumor principles via immunofluorescence (IF) and immunohistochemical (IHC) staining. HIF-1α-positive signals are drastically decreased after treatment with either MnO_2_-BSA or ^131^I-MnO_2_-BSA (Fig. 3i, j), validating that MnO_2_-BSA indeed reacts with H_2_O_2_ and induces H_2_O_2_ decomposition into O_2_ to successfully mitigate hypoxia.

Hypoxia mitigation also benefits immunosuppressive microenvironment modulation and reinforces immunity [34–36]. Consistently, MnO_2_-containing groups, i.e., G3 monotherapy and G4 combined therapy, are found to significantly augment the percentage of CTLs in comparison to G1, wherein the combined one outperforms other groups (Fig. 3i, k). This result implies that O_2_ release-mitigated hypoxia microenvironment is expected to significantly propel T cell infiltrations into RT-treated tumors. Additionally, we explored the influences of hypoxia mitigation arising from MnO_2_-BSA on regulatory T cells (Tregs, marker Foxp3) and TAMs (marker F4/80) because these cells serve as the immunosuppressive milieu to inhibit CTL infiltration and facilitate tumor progression [13, 37]. Previous studies have showed that hypoxic TME and radiation can promote the recruitment of Tregs into tumors and drive TAM differentiation into pro-tumorigenic M2 phenotype [34–36]. Intriguingly, both MnO_2_-BSA and ^131^I-MnO_2_-BSA treatments robustly decrease the percentage of Foxp3-positive Treg cells (Fig. 3i, l), resulting in a considerably-increased CTL/Treg ratio, especially in mice treated with ^131^I-MnO_2_-BSA (Fig. 3m).

Moreover, MnO_2_-BSA alone and ^131^I-MnO_2_-BSA drastically reduce the population of TAMs in the tumors (Fig. 3i, n). More significantly, the number of M2-phetotype TAMs (marker CD206) significantly declines after treatment with ^131^I-MnO_2_-BSA in comparison to G1 (control) and G3 (^131^I alone) groups, signifying that ^131^I-MnO_2_-BSA treatment enables M2-type TAMs to polarize into M1-type ones (Fig. 3i, o), which is consistent with a previous study [16].

### ^131^I-MnO_2_-BSA for reinforcing ICD activation

Subsequently, we explored whether such IRT radiosensitizers-unlocked radiogenetics could induce ICD after magnifying the systematic immune responses. Herein, calreticulin (CRT) and high mobility group box 1 (HMGB1) that are known as the typical hallmarkers of ICD were inspected [38, 39]. ^131^I-MnO_2_-BSA significantly up-regulates the expression of CRT on cell surface, suggesting that ^131^I-MnO_2_-BSA treatment indeed induced ICD. The overall expression of HMGB1 show no obvious alterations between G1 and either G3 or G4, which can be ascribed to that ^131^I-MnO_2_-BSA treatment merely allows HMGB1 to translocate from nucleus to cytosol (Additional file 1: Fig. S4a, c). Coincidently, all of these modulations can lead to the increased secretions and births of some pro-inflammatory cytokines such as interferon gamma (IFN-γ) and tumor necrosis factor alpha (TNF-α) that also play crucial roles in the cytotoxic functions of CTLs (Additional file 1: Fig. S4b, d). Moreover, ^131^I-MnO_2_-BSA treatment is found to slightly reduce the expression of IL10 secreted by M2 macrophages, which indirectly reflect M2-type TAMs decline and immunosuppressive microenvironment mitigation, which is in accordance with a previous report (Additional file 1: Fig. S4b, d) [24].

**Fig. 4:**
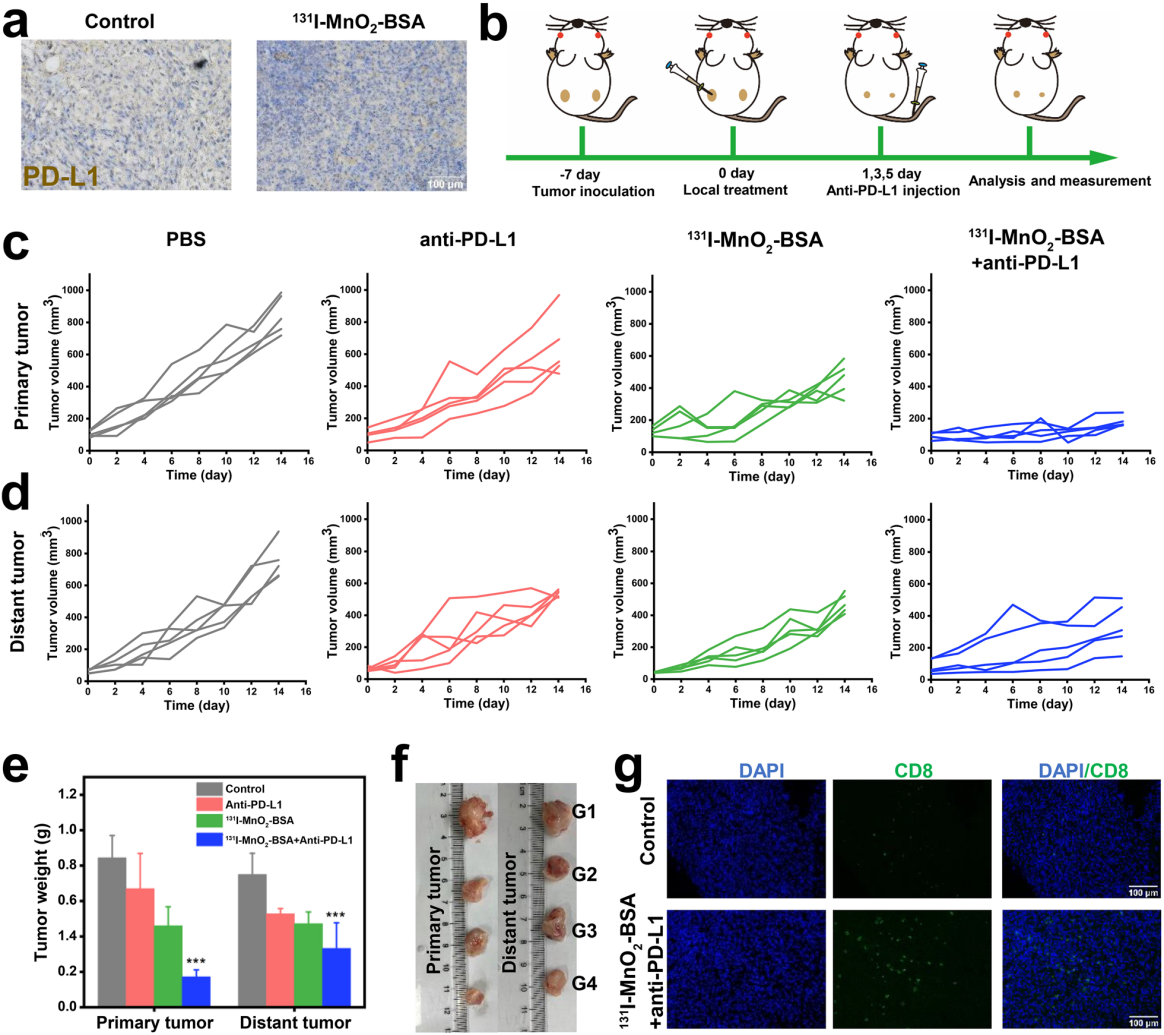
Combined IRT-ICB therapy for inhibiting metastatic tumor using such Mn-based radiosensitizers-activated systematic immune responses on the bilateral 4T1 tumor-bearing mice. **a** Immunohistochemical images of tumor slices to show the expression levels of PD-L1 on tumor cells collected from mice. **b** Schematic of experimental procedures on the abscopal bilateral 4T1 tumor model for IRT-ICB therapy. **c**, **d** Primary (**c**) and distant (**d**) tumor growth curves of mice after treatments with anti-PD-L1, ^131^I-MnO_2_-BSA and ^131^I-MnO_2_-BSA + anti-PD-L1. **e**, **f** Tumor weights (**e**) and representative digital photos (**f**) of primary and distant dissected tumors harvested from mice after 14 days post-various treatments (G1–G4). **g** Immunofluorescence images of primary and distant tumor slices stained with anti-CD8 antibody after 14 days post-corresponding treatments in both Control and ^131^I-MnO_2_-BSA + anti-PD-L1 groups. P values were calculated by ANOVA (***P < 0.001). Data are expressed as mean ± SD (*n* = 5). G1–G4 represent Control, Anti-PD-L1, ^131^I-MnO_2_-BSA and ^131^I-MnO_2_-BSA + Anti-PD-L1, respectively. Dose: 500 µCi

Unexpectedly, the expression of PD-L1 on 4T1 tumors is promoted by ^131^I-MnO_2_-BSA–unlocked radiogenetics compared to other groups (Fig. 4a). Immune ligand overexpression on tumor cells sensitizes these tumor cells to their related antibodies, and enhances the antitumor responses to related ICB-based antitumor immunity [40]. This phenomenon provides us a distinctive insight into the IRT-activated immune responses and inspires us to develop the combined therapy with ICB (i.e*.*, ^131^I-MnO_2_-BSA and anti-PD-L1) to repress distant tumors.

### Combined IRT with ICB to inhibit the distant tumors

Inspired by above radiogenetics-enabled PD-L1 up-regulation, IRT combination with ICB was expected to further generate DAMPs and TAAs and activate systematic immune responses to repress metastatic tumors. To assess the anti-metastatic effects, a bilateral 4T1 tumor-bearing mouse model was established to figure out whether the synergistic effect of ^131^I-MnO_2_-BSA and anti-PD-L1-based ICB can repress tumor metastasis via potentiating systematic immune responses (Fig. 4b).

Anti-PD-L1 therapy alone has been found to inhibit both the primary and distant tumors to some extent, but the antitumor outcomes are insufficient. Although local ^131^I-MnO_2_-BSA injection is able to destroy primary tumors, it has no obvious influence on distant tumors (Fig. 4c, d and Additional file 1: Fig. S5a, b). In contrast, combined IRT and anti-PD-L1-mediated ICB results in a considerably-elevated antitumor activity, which not only distinctly kills the primary tumor but also destroys the distant tumor (Fig. 4c, d and Additional file 1: Fig. S5a, b). The average tumor weight and the representative images of dissected tumor show that the tumor weight and volume in mice treated with ^131^I-MnO_2_-BSA plus anti-PD-L1 are much lower than those in other groups. These compelling evidences adequately indicate the excellent antitumor and anti-metastasis performance of IRT-ICB via activating immune memory effects (Fig. 4e, f). No appreciable body weight variations between either two groups imply no obvious side effects of this treatment strategy (Additional file 1: Fig. S5c). To determine the anti-metastasis mechanism, distant 4T1 tumors at control group and IRT-ICB groups were harvested, and the levels of infiltrated CTLs in distant tumors were examined by immunofluorescence staining. Result shows that the proportion of CTLs in ^131^I-MnO_2_-BSA plus anti-PD-1 group is much higher than that in control group, which uncovers that the immune memory effect-enabled CTLs infiltrations in distant tumors are responsible for the significantly-elevated anti-metastasis effects (Fig. 4g).

### Generality of such Mn-based radiosensitizers-unlocked radiogenetics

**Fig. 5:**
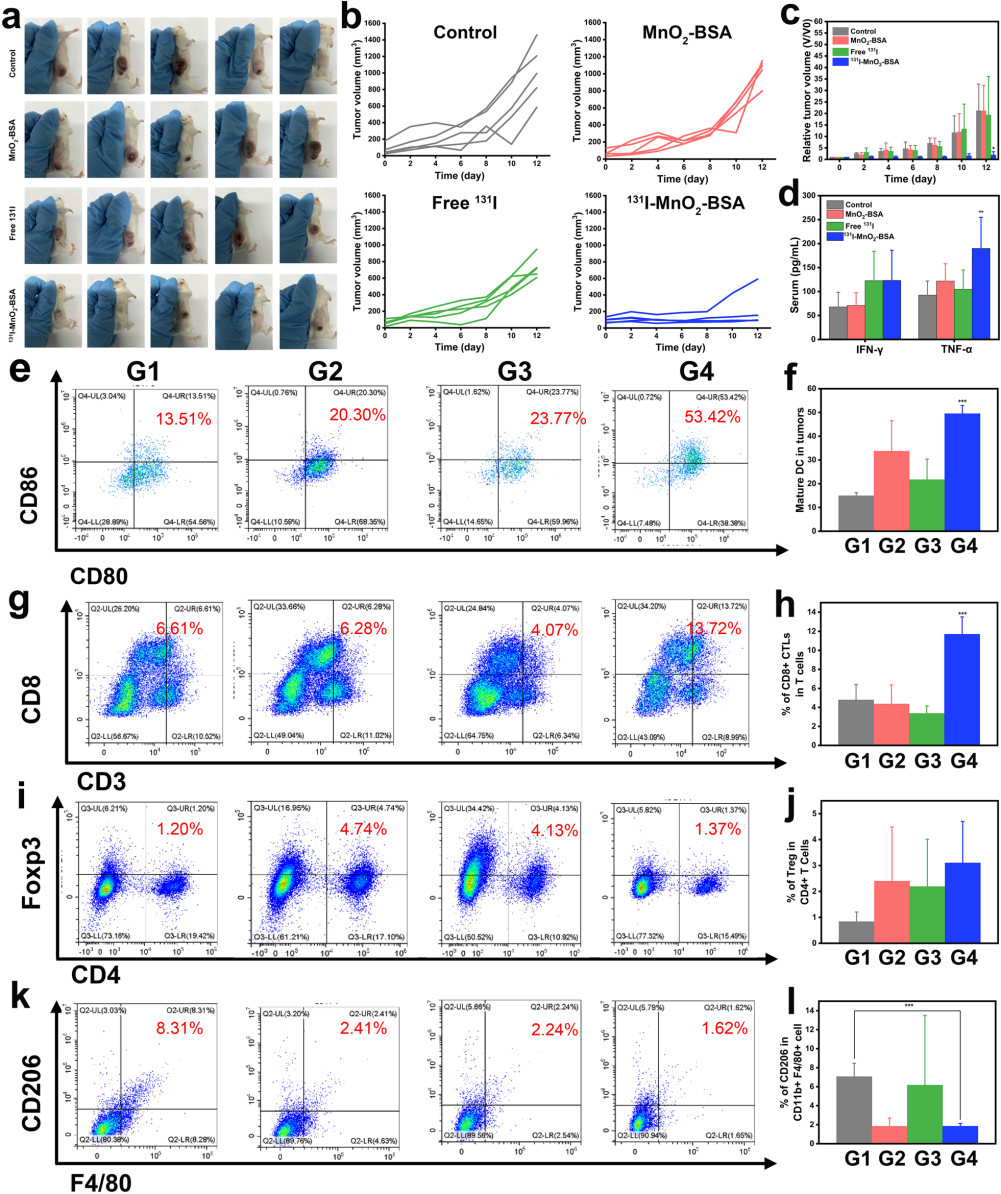
In vivo antitumor evaluations using ^131^I-MnO_2_-BSA on CT26-bearing mice model. **a** Digital photos of CT26 tumor-bearing mice that experienced different treatments at the end of experimental period, dose: 500 µCi; **b** Tumor growth curves of each mouse in different groups such as control, MnO_2_-BSA, free ^131^I and ^131^I-MnO_2_-BSA (dose: 500 µCi). **c** Time-dependent relative tumor volume variations of CT26 tumor-bearing mice experiencing corresponding treatments in different groups including control, MnO_2_-BSA, free ^131^I and ^131^I-MnO_2_-BSA (dose: 500 µCi), where tumor volumes were normalized to initial values (V/V0). Data are mean ± SD (*n* = 5). **d** ELISA-determined secretion levels of INF-γ and TNF-α in serum harvested from CT26-bearing mice that experienced different treatments with control, MnO_2_-BSA, free ^131^I and ^131^I-MnO_2_-BSA. **e**–**l** FCM patterns and corresponding statistical data of matured dendritic cells (CD80+CD86+) (**e**, **f**), CD3+CD8+CTLs (**g**, **h**), Tregs (**i**, **j**), CD206+CD11b+F4/80+(M2 macrophages) (**k**, **l**) in tumors harvested from CT26-bearing mice that experienced different treatments with G1–G4. Values are represented as mean ± SD (*n* = 3). G1–G4 represent Control, MnO_2_-BSA, free ^131^I and ^131^I-MnO_2_-BSA treatment, respectively. P values were calculated by ANOVA (**P < 0.01 and ***P < 0.001). Dose: 500 µCi

To validate the activated systematic immune responses produced by such Mn-based radiosensitizers-unlocked radiogenetics, another tumor model (i.e., CT26) was used, and results identical to those found in above 4T1 model were acquired. In detail, the Mn-based radiosensitizers show significant CT26 cell killing effects (Additional file 1: Fig. S6) and repress tumor growth without altering mouse body weights (Additional file 1: Fig. S7a). This is attributed to the activation of systematic immune responses especially after combining with anti-PD-L1 therapy (Fig. 5a–c), e.g., antitumor cytokine secretion elevation (Fig. 5d), matured antigen-presenting cell increase (Fig. 5e, f), CD8+ effector T infiltrations recruitment (Fig. 5g, h), and Tegs (Fig. 5i, j) and immunosuppressive M2-type macrophage (Fig. 5k, l) decreases. In the bilateral CT26 tumor-bearing mouse model, identical results are obtained, as such IRT radiosensitizers outperform other groups with respect to primary tumor recession (Additional file 1: Fig. S8a–e) without influencing mouse body weights (Additional file 1: Fig. S7b). More significantly, ^131^I-MnO_2_-BSA radiosensitizers also perform the best in repressing the distant (or metastatic) tumors (Additional file 1: Fig. S8b, d, f) since they successfully activate the systematic immune responses associated with the increases in CD45+ (Additional file 1: Fig. S9a, c), CD8+ (Additional file 1: Fig. S9b, d) and IFN-γ and TNF-α (Additional file 1: Fig. S9e) when combined with ICB. These compelling and remarkable results adequately validate the generality of such Mn-based radiosensitizers-unlocked radiogenetics.

In the biosafety evaluation, the negligible variations of liver and kidney function indexes suggest that this IRT-ICB combination therapy fails to induce liver or kidney dysfunctions (Additional file 1: Fig. S10). Moreover, no clinically meaningful changes are found between the IRT-ICB groups and control groups including routine blood and biochemical indicators (Additional file 1: Fig. S10). Significantly, histological analysis of the main organs reveals no gross pathological changes (Additional file 1: Fig. S11). Taken together, IRT-ICB treatment can be regarded as a potential remedy for clinical applications. These inspiring results denote that ^131^I-MnO_2_-BSA hold high clinical translation potential since their components (i.e., ^131^I and BSA) have been used in clinics and MnO_2_ also share high biosafety especially in acidic tumor microenvironment because of dissolution into Mn^2+^ [23, 24].

## Conclusions

We successfully constructed ^131^I-labeled Mn-based radiosensitizers featuring high labeling stability to open up radiogenetics as a method to repress tumor progression and metastasis, addressing the insufficient systematic immune response activation that current local treatment approaches suffer from. ^131^I-MnO_2_-BSA achieved high retention in tumors and displayed excellent therapeutic efficiency. Catalytical O_2_ release by MnO_2_ from the ^131^I-MnO_2_-BSA radiosensitizers not only alleviated hypoxic microenvironment, but also remodeled the immunosuppressive microenvironment by increasing CTLs recruitment, blocking Tregs and TAMs infiltrations, promoting M2-type TAMs polarization and secreting more antitumor cytokines. Additionally, RNA sequencing validated that the antitumor mechanism correlated with hypoxia and immune response-underlined tumor microenvironment. More significantly, such IRT radiosensitizers-unlocked radiogenetics also upregulated PD-L1, which allowed radioimmunotherapy represented by ^131^I-MnO_2_-BSA plus anti-PD-L1 blockade to exert high antimetastatic activity by activating systematic immune responses on both 4T1 and CT26 mouse models. Collectively, the Mn-based radiosensitizers-unlocked radiogenetics described here that can magnify the systematic immune responses of local IRT by reshaping hypoxic and immunosuppressive tumor microenvironment and serve as a general method to guide new radiosensitizers development.

## Materials and methods

### Materials

Bovine serum albumin (BSA), Manganese chloride tetrahydrate (MnCl_2_·4H_2_O), and sodium hydroxide (NaOH) were from Sigma-Aldrich. Roswell park memorial institute (RPMI) 1640 medium, penicillin–streptomycin, fetal bovine serum (FBS) were obtained from Gbico. The cell counting kit-8 (CCK-8), DCFH-DA probe, and DAPI assay kit were obtained from Beyotime Biotechnology Co., Ltd. Annexin V-FITC apoptosis detection kit was purchased from BD Pharmingen. Na^131^I solution was bought from Shanghai Xinke Pharmaceutical Co., Ltd. (Shanghai, China). Ultrapure water (Milli-Q, Millipore, Bedford, MA, USA) was used throughout the experiment.

### Synthesis of MnO_2_-BSA nanoparticles

The MnO_2_-BSA were synthesized following a BSA-constrained biomimetic method. BSA (125 mg) was dissolved in 50 mL of purified water, followed by addition of MnCl_2_·4H_2_O solution (0.1 M, 250 μL). Then, the mixture was stirred at 37 °C for 3 min. Then, 500 μL of NaOH solution (1 M) was used to adjust the pH value to 11 ~ 12 and concurrently the mixture color was changed into brown. After 6 h reaction at 37 °C, the solution was dialyzed (MWCO = 8000 ~ 14,000 Da) against deionized water for 24 h to remove redundant Mn^2+^. Ultimately, the powder was collected after lyophilization and stored at 4 °C for further experiments.

### Characterization of ^131^I-MnO_2_-BSA

The morphology and size of ^131^I-MnO_2_-BSA was characterized by transmission electron microscope (Tecnai G2 F20 S-TWIN, Netherlands). The size distribution of ^131^I-MnO_2_-BSA were measured by dynamic light scattering (DLS) using a Malvern Zetasizer Nano ZS instrument. Absorption spectra of BSA and ^131^I-MnO_2_-BSA were acquired by an UV–Vis spectrophotometer (UV-2450, Shimadzu, Japan). The X-ray photoelectron spectroscopy (XPS) analysis was performed to determine the elemental composition of nanoparticles on the Thermo Scientific™ K-Alpha™^+^ spectrometer. The samples were quantified on the inductively coupled plasma-optical emission spectrometry (ICP-OES, Agilent 730).

### In vitro O_2_ generation

O_2_ generation from ^131^I-MnO_2_-BSA NPs was measured in a sealed chamber coupling with an oxygen electrode (Dissolved Oxygen Meter, AR-8010, China) at 37 °C. ^131^I-MnO_2_-BSA NPs (180 μM Mn) were dispersed in PBS, and the pre-dissolved O_2_ was removed by bubbling with N_2_ for 30 min. H_2_O_2_ (100 μM) was then injected into the chamber and the generated O_2_ was recorded at the pre-set time points.

### Radioisotope labeling and labeling stability assay

MnO_2_-BSA was labeled with ^131^I using a standard chloramine-T oxidation method according to previous protocol [41]. MnO_2_-BSA (1 mg mL^−1^, 1 mL), Na^131^I (1 mCi) and chloramine-T (100 μg) were mixed together and reacted for 10 min at room temperature. The reacted solution was collected and purified using an Amicon filters (MWCO = 30 kDa) to remove excessive ^131^I until no detachable gamma activity in the filtrate solution. For testing the labeling capacity and stability, Whatman No.1 filter paper was used and detected by γ-detector. The in vitro stability of ^131^I labeled MnO_2_-BSA were tested by co-culturing with PBS, 1640 medium and serum (100 μL for each) at 37 °C and measured at 0 h, 4 h, 12 h and 24 h, respectively.

### Cytotoxicity assay

The 4T1 murine breast cancer cell line and CT26 murine colon cell line were originally obtained from American Type Culture Collection (ATCC) and cultured under recommended conditions. The cells were cultured in RPMI-1640 medium plus 10% fetal bovine serum and incubated at 37 °C under 5% CO_2_ in a cell incubator. 4T1 cells and CT26 cells were seeded in 96-well plates with a density of 5000 cells per well overnight to allow cell adherence. MnO_2_-BSA, free ^131^I, ^131^I-MnO_2_-BSA were co-cultured with 4T1 cells and CT26 cells at different concentrations for 48 h, respectively. Then, their viabilities were evaluated according to the CCK8 assay kit protocol for determining in vitro cytotoxicity.

### In vitro ROS generation

After co-culturing with MnO_2_-BSA (90 µM), free ^131^I (500 μCi mL^−1^), and ^131^I-MnO_2_-BSA (500 μCi mL^−1^) for 24 h, the cellular-ROS level was monitored by the ROS Assay Kit (Beyotime Biotechnology). After washing with PBS, the cells were stained with DCFH-DA (10 μM) for 30 min. Finally, the in vitro ROS generation ability was detected by the flow cytometry (FCM). Data were processed by FlowJo.

### Flow cytometry for determining cell apoptosis

Flow cytometry was used to analyze cell apoptosis. 4T1 cells were seeded in six-well plates and permitted to adhere overnight. MnO_2_-BSA (90 µM), free ^131^I (500 μCi mL^−1^) and ^131^I-MnO_2_-BSA (500 μCi mL^−1^) were incubated with 4T1 cells for 24 h. Then, the cells were digested and collected by centrifugation at 1500 rpm. Subsequently, the annexin V-FITC apoptosis detection kit was utilized to stain the cells according to the instructions before flow cytometry analysis.

### Tumor model establishment

All animals were provided by the animal center of Shanghai Tenth People’s Hospital, and all animal experiments were approved by Animal Welfare Ethics Committee of Shanghai Tenth People’s Hospital with an approval number (ID: SHDSYY-2020–074). To construct the tumor-bearing mice model, 4T1 cells or CT26 cells (1 × 10^6^) were subcutaneously injected into the back of BALB/c nude mice. In order to saturate the thyroid and decrease the uptake of thyroid, mice were administrated with potassium iodide solution (1%) for 7 days.

### In vivo tumor imaging

For intratumoral drug metabolism, SPECT/CT scanning (GE discovery 670, USA) was conducted so as to mimic clinical circumstance. The mice were firstly anesthetized with 3% pentobarbital through intraperitoneal injection and then imaged at different time intervals (4 h, 15 h, 24 h, 36 h, 48 h, 168 h) post-injection, wherein potassium iodide (1%) was used to block the thyroid uptake of the desseminated ^131^I-MnO_2_-BSA.

### Local IRT

Mice bearing subcutaneous 4T1 tumors or CT26 tumors were randomly divided into four groups for the following treatments: G1: control, G2: MnO_2_-BSA (*i.t.*, 90 µM), G3: free ^131^I (*i.t.*, 500 µCi), G4: ^131^I-MnO_2_-BSA (*i.t.*, 500 µCi), respectively. Body weight and tumor dimensions including length and width of each mouse were measured once per two days, according to which the tumor volume was obtained using the following equation: tumor volume = width^2^ × length/2. Relative tumor volumes were calculated as V/V_0_ (V_0_ was the tumor volume at the beginning of experiment). After 12 days post-treatment, 4T1 tumor tissues were collected from the BALB/c mice of each group for H&E staining and TUNEL staining.

### In vivo RNA-seq analysis

RNA extraction, quality control, library construction and sequencing were performed as previous procedures by BGI company. Differentially expressed genes (DEGs) were calculated with DESeq2 R package [42]. The *P* < 0.05 and fold change |log2 fold change|> 0.9 were set as the cutoff criteria. Volcano plot was drawn with sangerbox, and heatmap plot was performed with TB tools [43]. GO enrichment analysis were conducted with DAVID (DAVID, https://david.ncifcrf.gov/) and visualized by the R package “ggplot2” [44]. Significant enrichments were identified when the *P*-values were less than or equal to 0.05. The Search Tool for the Retrieval of Interacting Genes (STRING, https://string-db.org/) database was used to build the protein–protein interaction networks and visualized by Cytoscape.

### Immunofluorescence and immunohistochemical staining of harvested tumor slices after ^131^I-MnO_2_-BSA IRT

HIF-1α was detected to evaluate hypoxia by immunofluorescence staining. Besides, to evaluate the influence of ^131^I-MnO_2_-BSA on infiltrations of immune cells including CTLs, macrophage cells, M2-type macrophages and Tregs, tumors were stained with anti-CD8, anti-F4-80, anti-CD206, and anti-Foxp3 antibodies according to their vendor’s protocols, respectively. Immunofluorescence images were collected and analyzed by ImageJ software. Then, the expression of CRT and HMGB1 were examined and evaluated by immunohistochemical stainings. Besides, immune cytokines including interferon-gamma (IFN-γ), tumor necrosis factor (TNF-α), interleukin-10 (IL-10) and PD-L1 were also detected and evaluated.

### In vivo antitumor immunity

To explore the antitumor immune effect, CT26 tumors were collected and digested to prepare single cell suspensions. The cell suspensions were stained with the corresponding antibodies to identify the matured DCs (FITC-anti-mouse CD11c, PE-anti-mouse CD86, APC-anti-mouse CD80 antibodies), the activated T cells (FITC-anti-mouse CD3 and APC-Cyanine7-anti-mouse CD8 antibodies) and Tregs (BV421-anti-mouse CD4 and PE-anti-mouse Foxp3 antibodies), or macrophages (APC-Cyanine7-anti-mouse F4/80 and FITC-anti-mouse CD11b antibodies for total macrophages, and PE-anti-mouse CD206 antibodies for the M2-phenotype macrophages), followed by flow cytometry analysis. In addition, interferon gamma (IFN-γ) and tumor necrosis factor alpha (TNF-α) in serum were determined by enzyme-linked immunosorbent assay (ELISA) assay (Multi sciences).

### Radioisotope-immunotherapy for suppressing distant tumors

For distant tumors inhibition, 4T1 cells and CT26 cells were inoculated onto both flanks of each BALB/c mouse. Mice were administrated with potassium iodide solution (1%) for 7 days before corresponding treatments. The mice bearing tumors were divided into four groups randomly (G1: control, G2: anti-PD-L1, G3: ^131^I-MnO_2_, G4: ^131^I-MnO_2_ + anti-PD-L1). Herein, the clone of the anti-PD-L1 antibody used is 10F.9G2, which can react with murine PD-L1. For left flank tumor, ^131^I-MnO_2_ (500 µCi) was intratumorally (*i.t*.) injected in both G3 and G4. Mice in G2 and G4 were then i.v. injected with anti-PD-L1 antibodies (20 µg per mouse) at 1, 3 and 5 days, respectively. After that, the left/right flank tumor sizes and body weight of each mouse were monitored once per two days. At the end of treatment, mice were euthanized, and distant 4T1 tumors were harvested to evaluate the CD8^+^T cells by staining with fluorescence-labeled anti-CD8 antibody. Besides, the major organs were also collected for H&E staining. Isolated distant CT26 tumors were digested to analyze the activated T cells (FITC-anti-mouse CD3 and APC-Cyanine7-anti-mouse CD8 antibodies). The concentration of IFN-γ and TNF-α in the serum were analyzed with ELISA assay (Multi sciences).

### In vivo toxicity evaluation

Blood samples were collected for biochemistry (serum) and blood routine (whole blood) analyses. At the same time, organ samples including lungs, heart, liver, spleen and kidneys, were harvested and rinsed three times with PBS, followed by fixing with tissue fixative. Then, all tissues were embedded in paraffin and sectioned at 3-μm thickness, followed by H&E staining using a standard protocol and finally observed under a microscope.

### Statistical analysis

The statistical analysis carried out with Origin 2019. All experimental data was expressed in this manuscript as mean ± standard deviation. The statistical significance of the differences was determined by one-way ANOVA. The asterisks indicate significant differences (* means *P* < 0.05, ** means *P* < 0.01, *** means *P* < 0.001).

## Supplementary Information

**Additional file 1.** Additional figures.

## Data Availability

The data of this study is available from the corresponding authors on reasonable request.

## Abbreviations

IRT: Interstitial radiotherapy
EBRT: External beam radiotherapy
ICD: Immunogenic cell death
ICB: Immune checkpoint blockade
Treg: Regulatory T cells
ROS: Reactive oxygen species
TAMs: Tumor associated macrophages
CTLs: Cytotoxic T lymphocytes
IFN-γ: Interferon-gamma
TNF-α: Tumor necrosis factor
IL10: Interleukin-10
DEGs: Differentially expressed genes
CRT: Calreticulin;
HMGB1: High mobility group box 1
