# Supplementary material for: Biomimetic radiosensitizers unlock radiogenetics for local interstitial radiotherapy to activate systematic immune responses and resist tumor metastasis
Source: J Nanobiotechnology. 2022 Mar 4;20:103. doi: 10.1186/s12951-022-01324-w (PMC8895626; doi:10.1186/s12951-022-01324-w)
Supplement: Supplementary file 1 — Additional file 1. Additional figures. [file 12951_2022_1324_MOESM1_ESM.doc]

# Biomimetic Radiosensitizers Unlock Radiogenetics for Local Interstitial Radiotherapy to Activate Systematic Immune Responses and Resist Tumor Metastasis

Jiajia Zhang,^1,2,#^ Mengdie Yang,^1,2,#^ Xin Fan,^1,2^ Mengqin Zhu,^1,2^ Yuzhen Yin,^1,2^ Hongyan Li,^3^ Jie Chen,^3^ Shanshan Qin,^1,2^ Han Zhang,^1,2^,* Kun Zhang,^2,3^,* Fei Yu^1,2^,*

**Supplementary Figures**


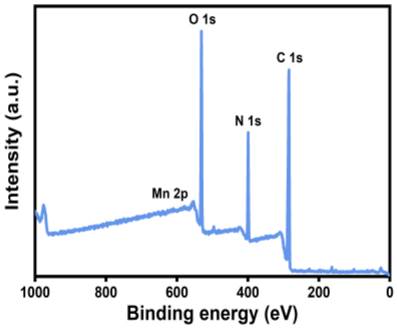


**Figure S1** Wide-band XPS spectrum of ^131^I-MnO_2_-BSA for determing Mn2p, N1s, O1s and C1s.


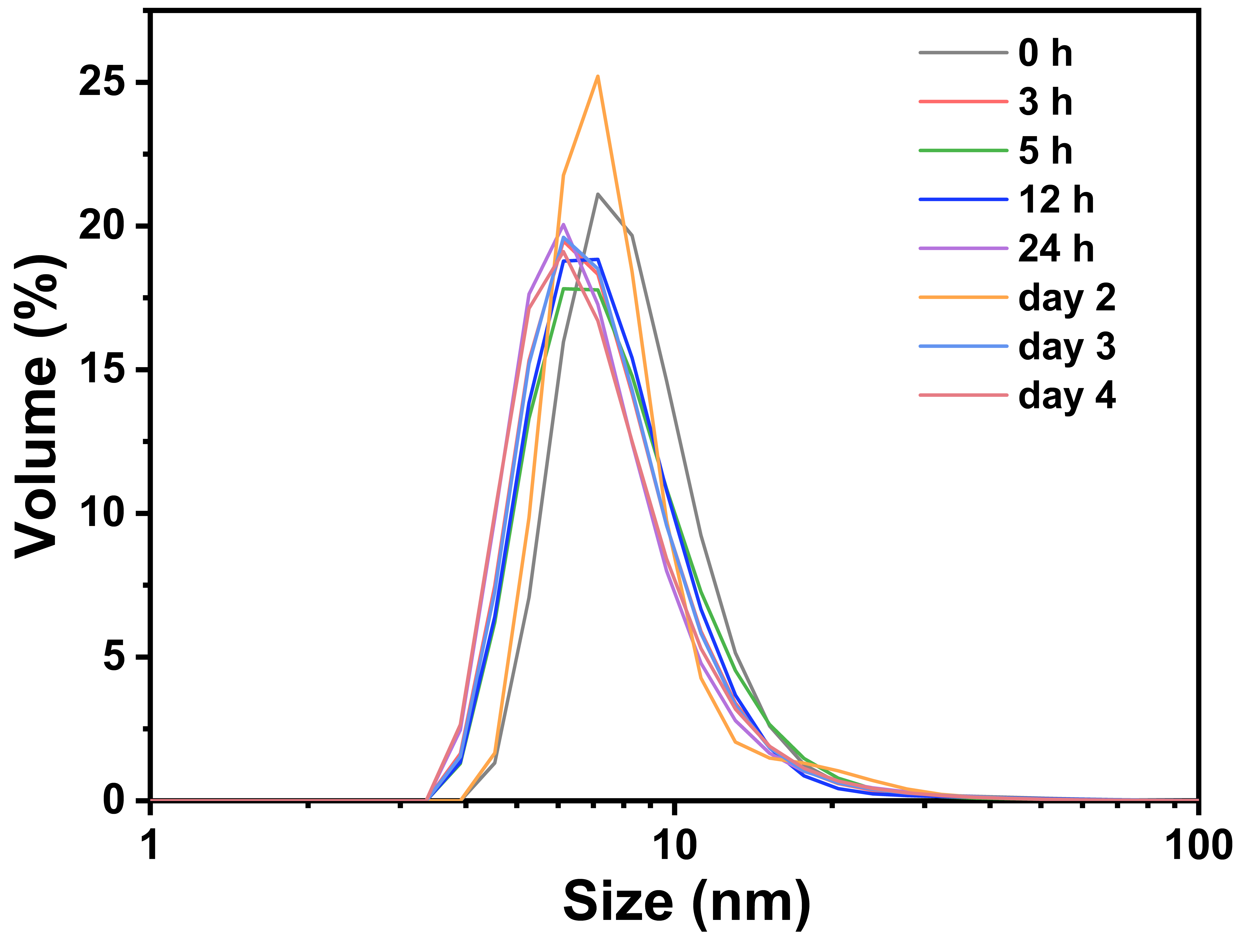


**Figure S2** Particle size distributions of ^131^I-MnO_2_-BSA in serum for different incubation time.


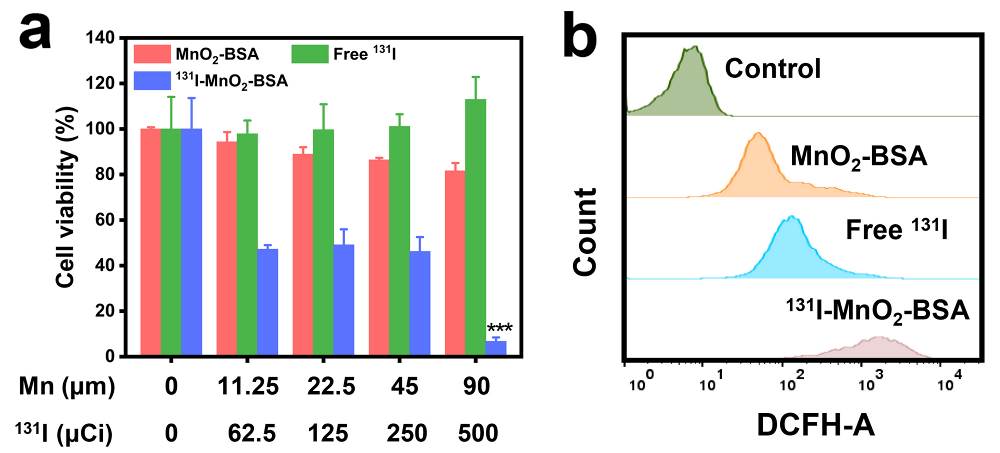


**Figure S3** (a) Cytotoxicity of MnO_2_-BSA, free ^131^I and ^131^I-MnO_2_-BSA against 4T1 cells at various concentrations at 48 h; (b) FCM analysis of ROS generation in 4T1 cells stained with DCFH-DA after various treatments.


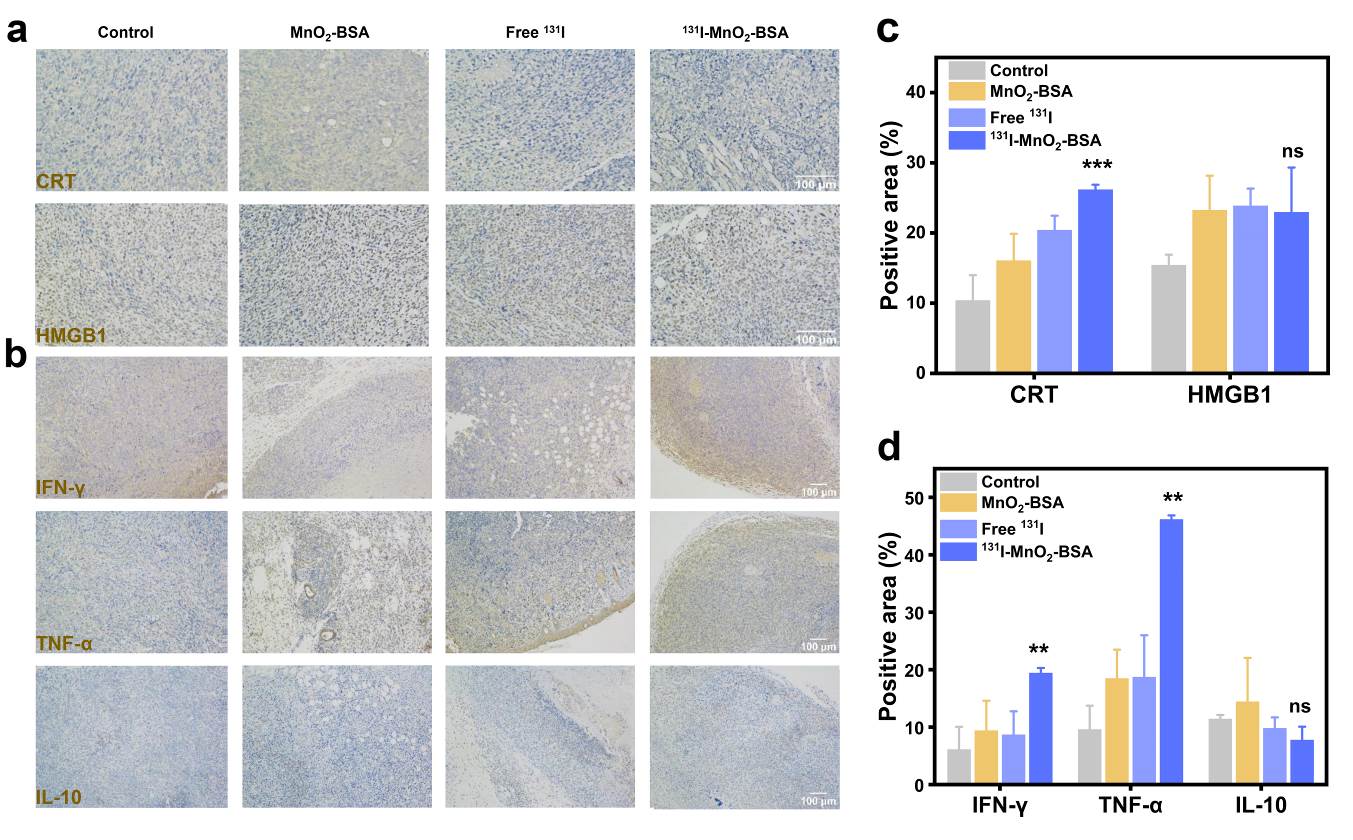


**Figure S4 ICD activation in IRT.** (a) Immunohistochemical examinations of CRT and HMGB1 after different treatments in 4T1 tumor model. (b) Typical immunohistochemical images of cytokines in tumors collected from mice after different treatments; (c) Statistical data of CRT-positive area and HMGB1-positive area, which were obtained from a; (d) Statistical data of IFN-γ-positive area, TNF-α-positive area, and IL-10-positive area, which were obtaind from b. P values were calculated by ANOVA (***P < 0.001; *P < 0.05; ns, not significant). Data are expressed as mean ± SD (n=3), dose: 500 µCi.


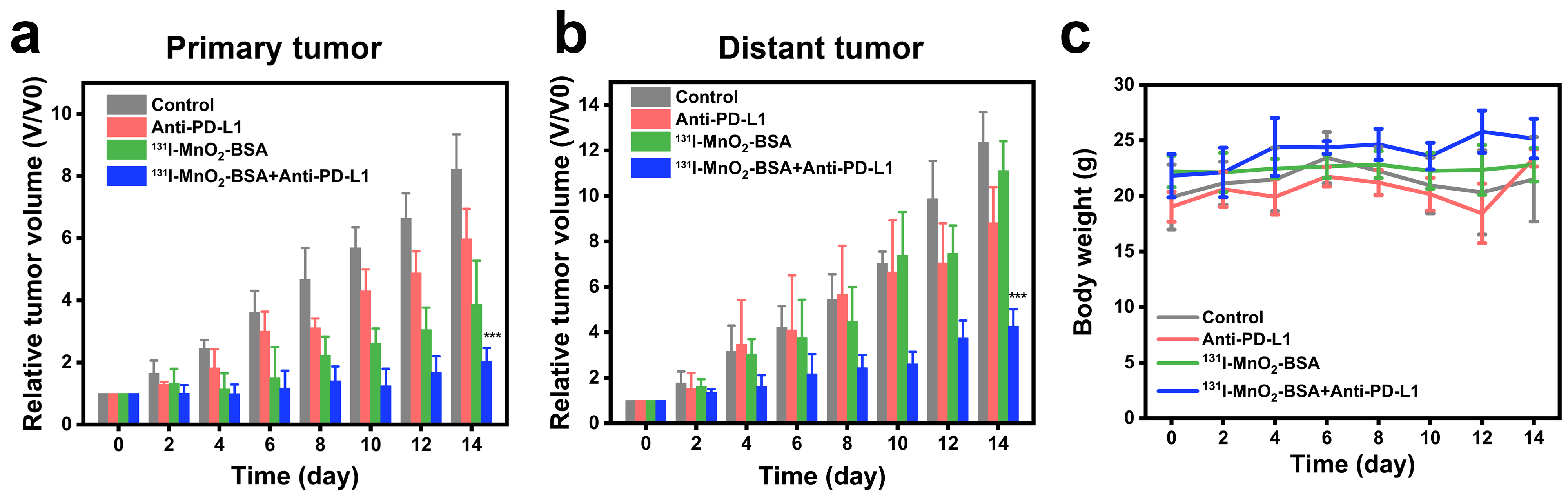


**Figure S5** (a,b) Primary (a) and distant (b) tumor growth curves after various treatments on bilateral 4T1-bearing mice model, and the relative tumor volumes were determined *via* normalization to initial values (V/V0). (c) Body weight variation of the bilaterall 4T1 tumors-bearing Balb/c mice during treatment.





**Figure S6** Cytotoxicity of MnO_2_-BSA, free ^131^I and ^131^I-MnO_2_-BSA against CT26 cells at various concentrations at 48 h.


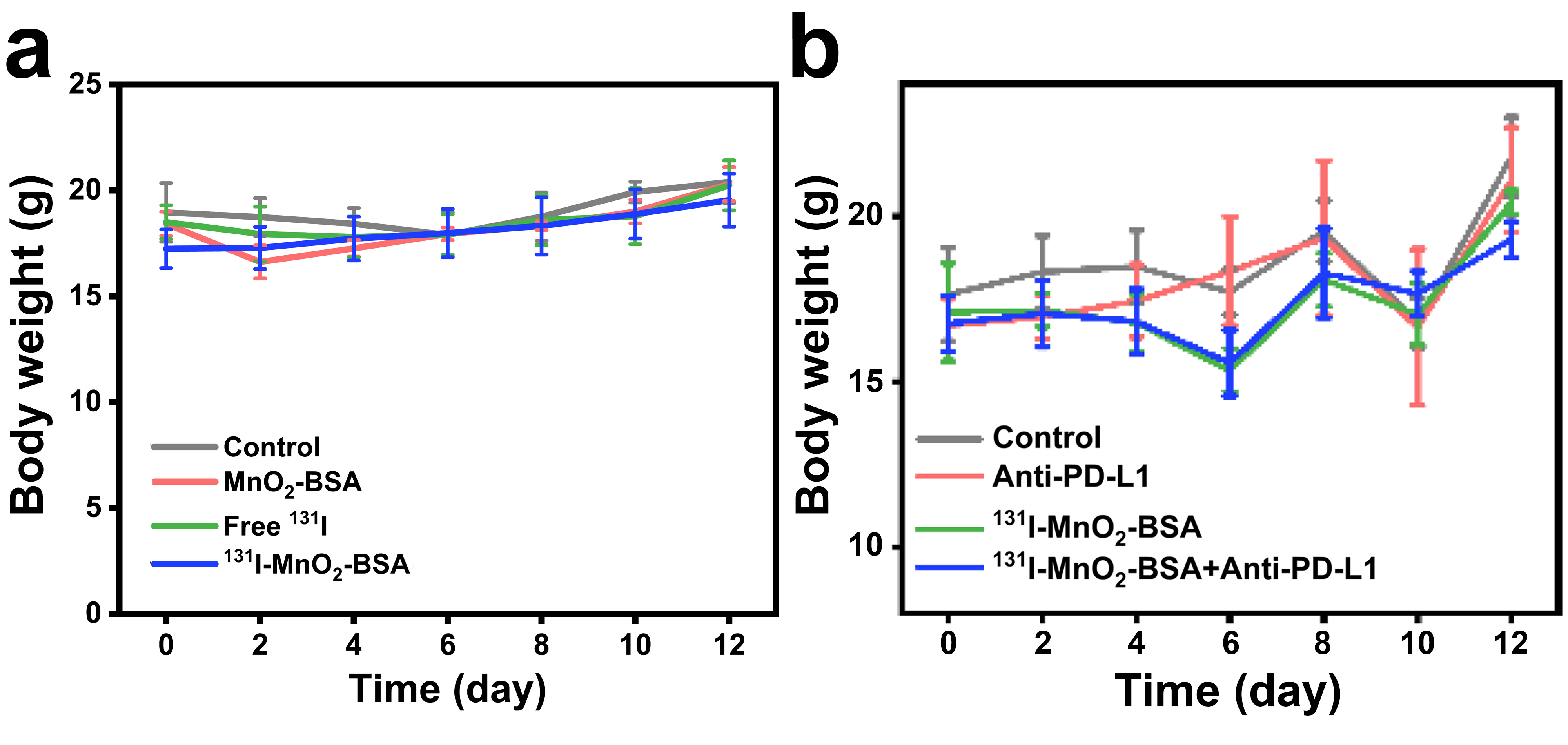


**Figure S7** (a,b) Body weight variations of CT26 tumors-bearing Balb/c mice (a) and bilaterall CT26 tumors-bearing Balb/c mice (b) during different treatments.


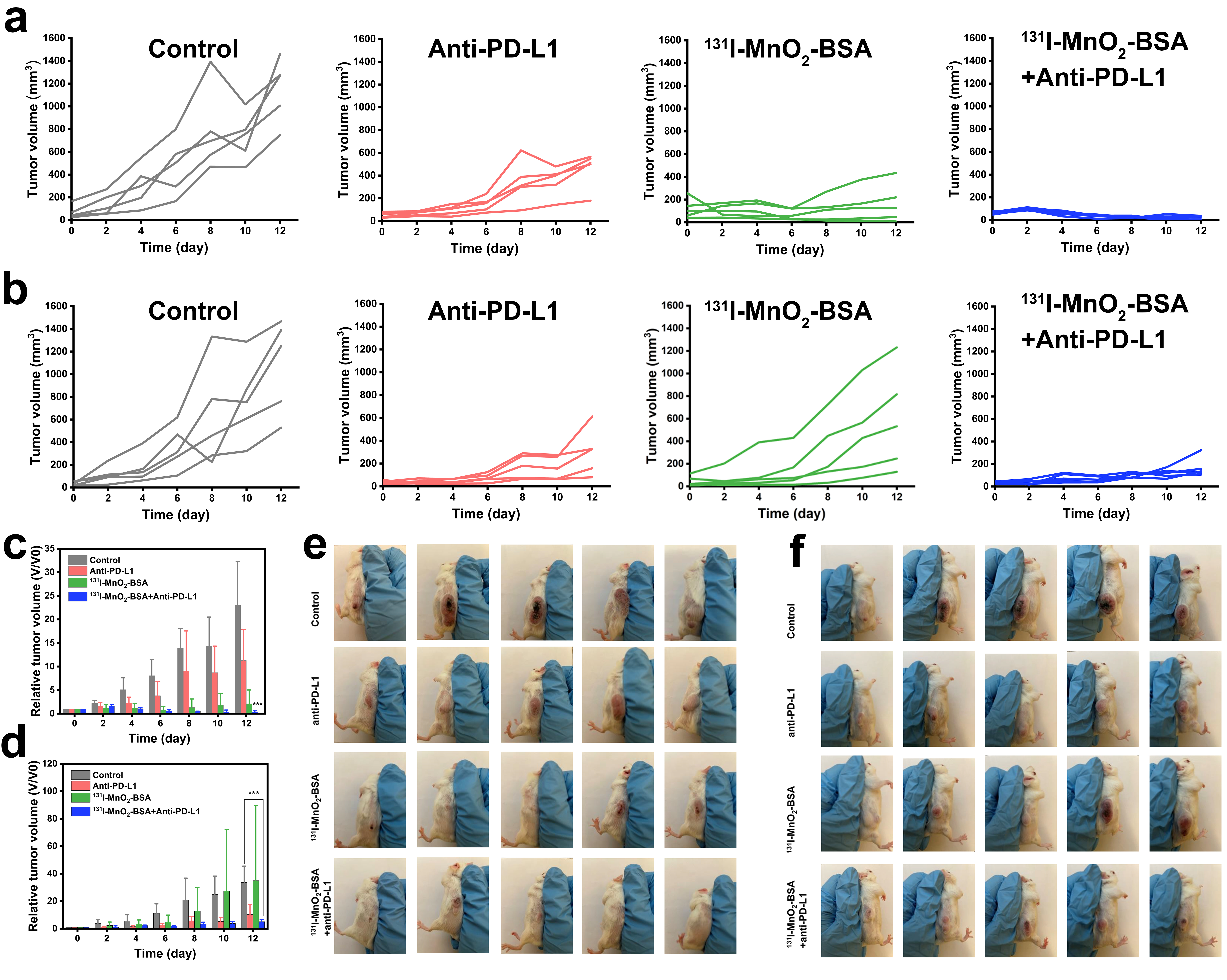


**Figure S8 Anti-tumor evaluations using such Mn-based radiosensitizers-unlocked radiogenetics for repressing tumor metastasis on bilateral CT26-beating mice model *via* activating systematic immune responses.** (a,b) Primary (a) and distant (b) tumor growth curves in each mouse after treatments with anti-PD-L1, ^131^I-MnO_2_-BSA and ^131^I-MnO_2_-BSA + Anti-PD-L1. (c,d) Time-dependent relative tumor volume variations of primary (c) and distant (d) tumors implanted on bilateral CT26 tumor-bearing mice experiencing corresponding treatments in different groups. (e,f) Digital photos of primary (e) and distant (f) tumors implanted on bilateral CT26 tumor-bearing mice that experienced different treatments at the end of experimental period (day 12), dose: 500 µCi. P values were calculated by ANOVA (***P < 0.001). Data are expressed as mean ± SD (n=5). Dose: 500 µCi.


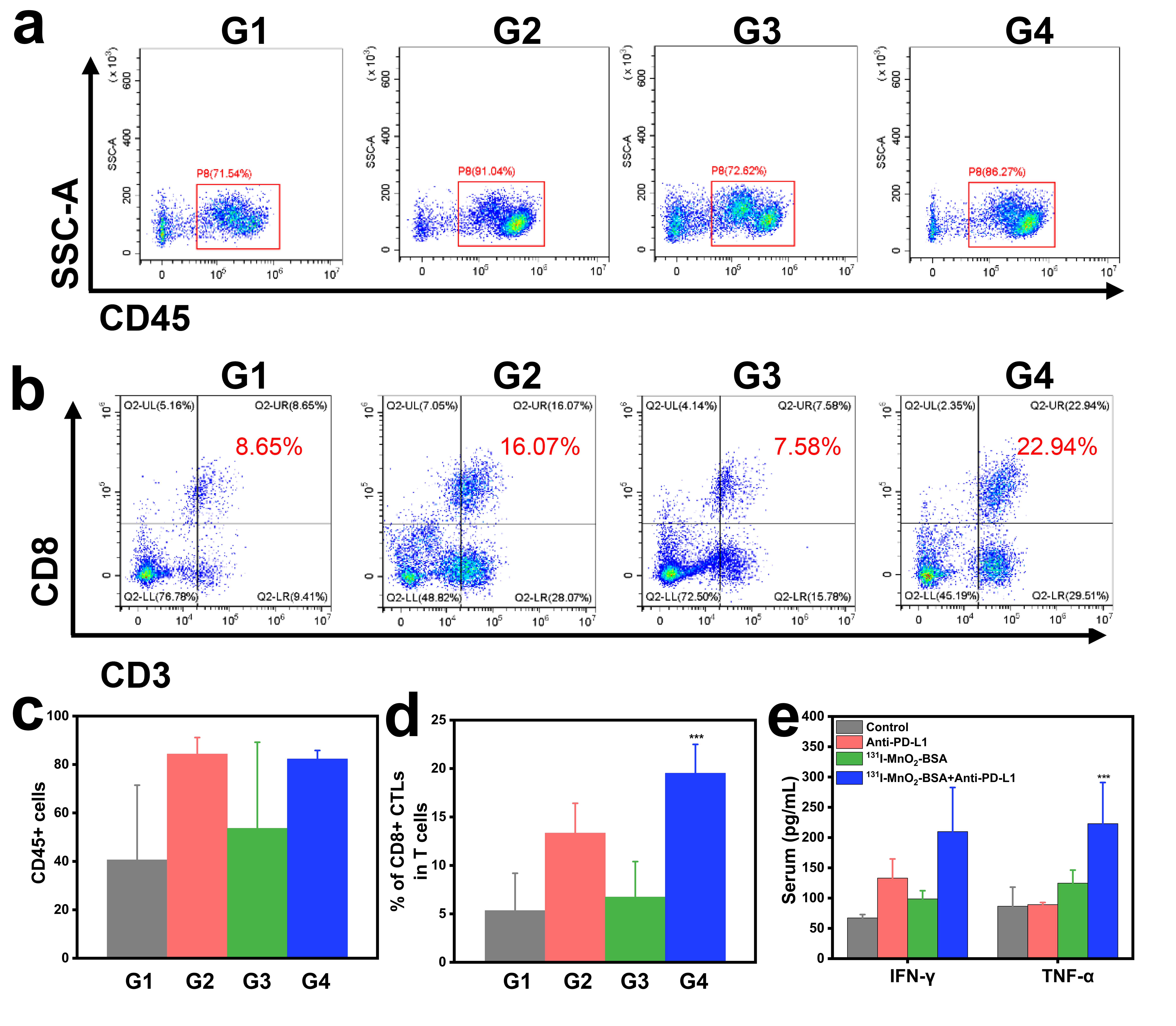


**Figure S9 Immune response activations using such Mn-based radiosensitizers-unlocked radiogenetics on bilateral CT26-beating mice model.** (a-d) FCM patterns (a,b) and corresponding statistical results (c,d) of CD45+ (a,c) and CD8+CD3+ CTLs (b,d) in distant tumors harvested from bilateral CT26-beating mice that experienced different corresponding treatments in G1-G4. (e) ELISA-determined secretion levels of INF-γ and TNF-α in serum harvested from bilateral CT26-bearing mice that experienced different treatments with G1-G4. Note: G1-G4 represent Control, Anti-PD-L1, ^131^I-MnO_2_-BSA and ^131^I-MnO_2_-BSA + Anti-PD-L1, respectively. Dose: 500 µCi. P values were calculated by ANOVA (***P < 0.001). Data are expressed as mean ± SD (n=5).

**
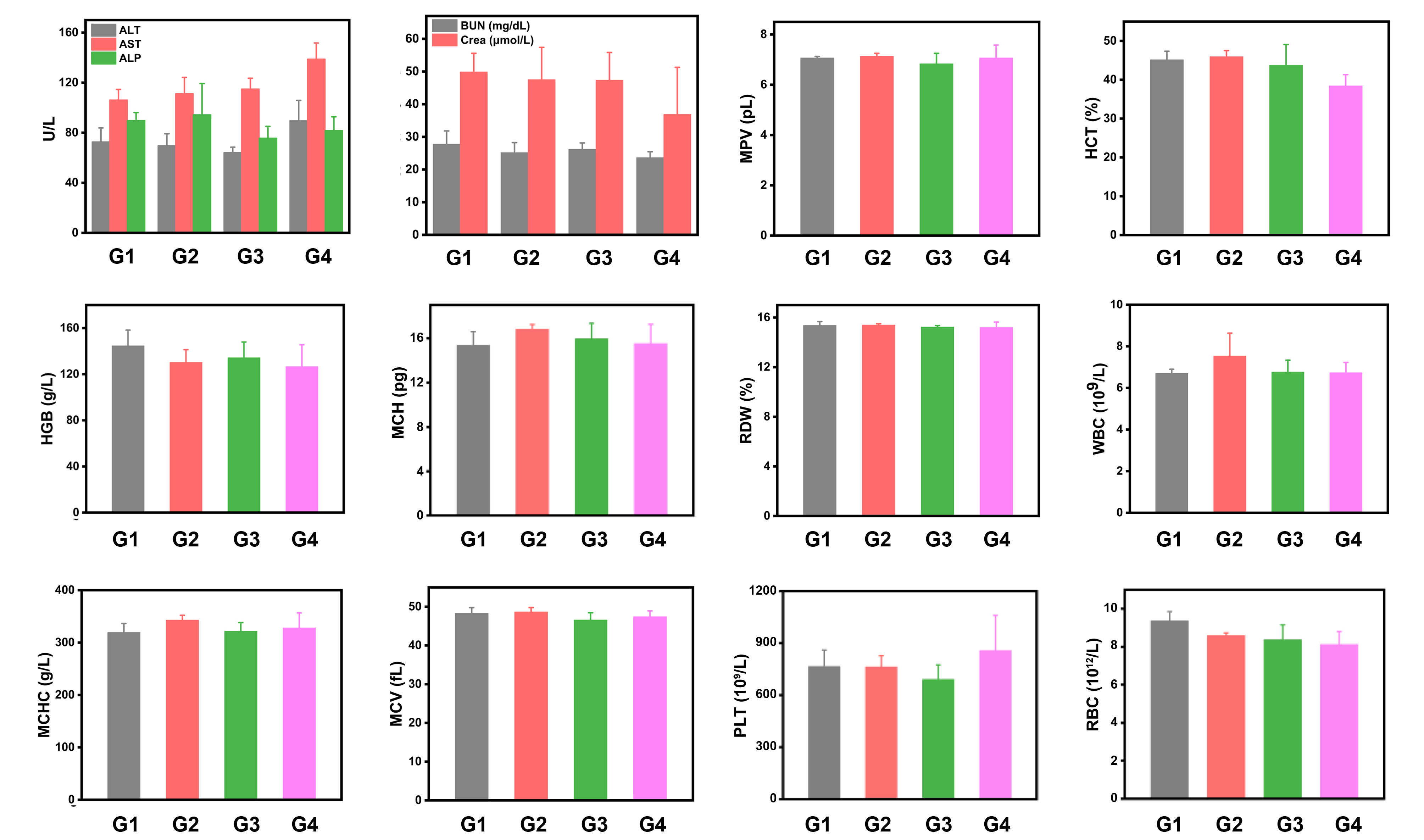
**

**Figure S10 *In vivo* biosafety evaluation post-different treatments with G1-G4,** including liver (*e.g.*, ALT, AST, ALP) and renal (BUN, Crea) function indexes analysis, and hematology analysis (MPV, HCT, HGB, MCH, RDW, WBC, MCHC, MCV, PLT and RBC). Note: G1-G4 represent Control, anti-PD-1, ^131^I-MnO_2_-BSA and ^131^I-MnO_2_-BSA+ anti-PD-1 treatment, respectively. Dose: 500 µCi.


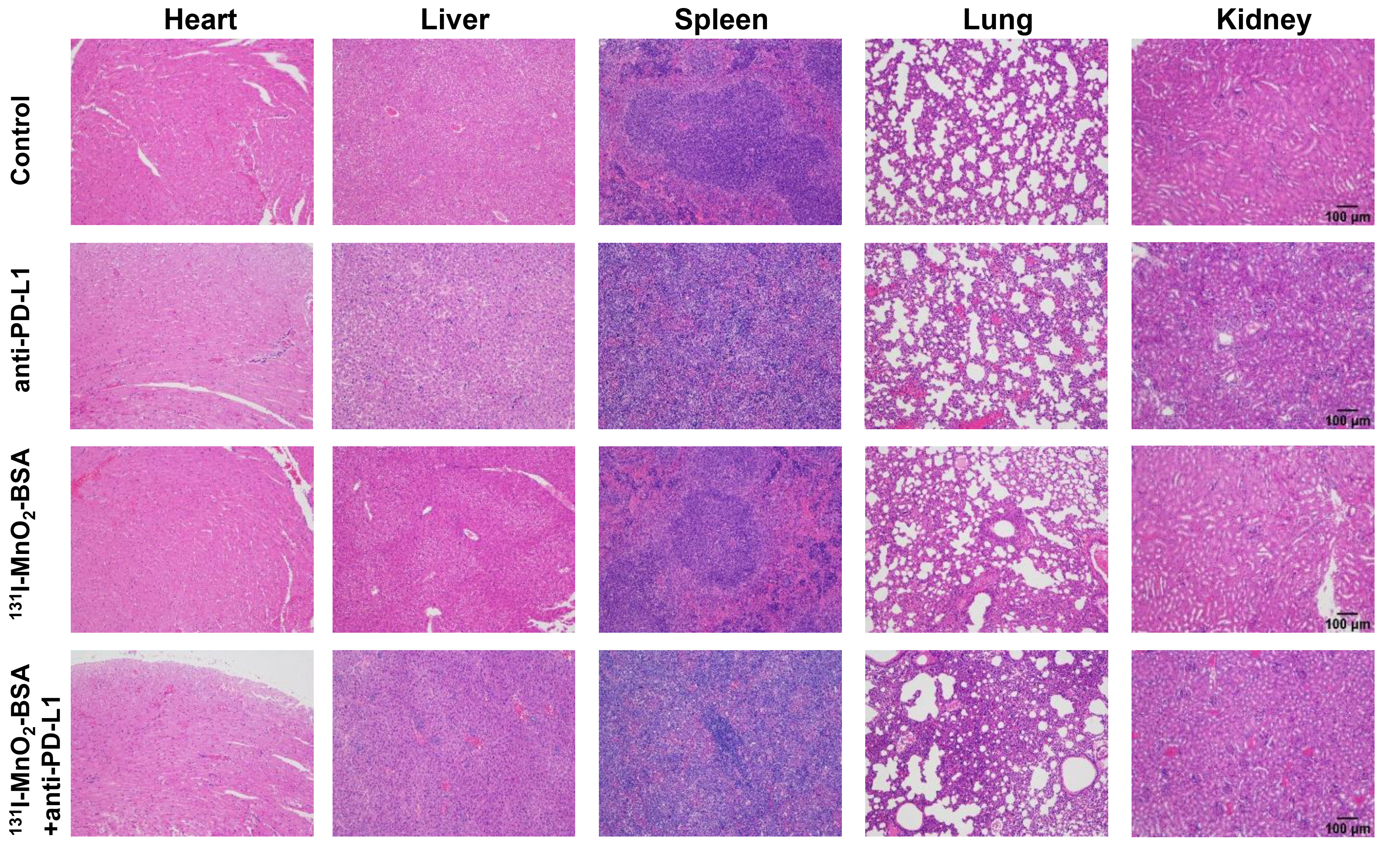


**Figure S11 *In vivo* biosafety evaluation post-different treatments with G1-G4,** including H&E-stained pathological inspections of normal organs (*e.g.*, heart, liver, spleen, lung and kidney). Note: G1-G4 represent Control, anti-PD-1, ^131^I-MnO_2_-BSA and ^131^I-MnO_2_-BSA+ anti-PD-1 treatment, respectively. Dose: 500 µCi.
